# Assessment of Regulatory T Cells in Childhood Immune Thrombocytopenic Purpura

**DOI:** 10.1155/2013/143687

**Published:** 2013-11-05

**Authors:** Karina L. M. Mazzucco, Lauro M. Junior, Natália E. Lemos, Andréa Wieck, Annelise Pezzi, Alvaro M. Laureano, Bruna Amorin, Vanessa Valim, Lucia Silla, Liane E. Daudt, Paulo J. C. Marostica

**Affiliations:** ^1^Programa de Pós-Graduação da Criança e do Adolescente, Universidade Federal do Rio Grande do Sul (UFRGS), Escola de Medicina, Porto Alegre, RS, Brazil; ^2^Departamento de Oncologia e Hematologia, Hospital da Criança Santo Antônio-Santa Casa de Misericórdia de Porto Alegre, Porto Alegre, RS, Brazil; ^3^Centro de Terapia Celular, Centro de Pesquisa Experimental, Hospital de Clínicas de Porto Alegre, RS, Brazil; ^4^Departamento de Pediatria da Escola de Medicina, Universidade Federal do Rio Grande do Sul, Porto Alegre, RS, Brazil

## Abstract

This study had the objective to assess the frequency of Tregs in children newly diagnosed with ITP and ascertain whether an association exists between Tregs and platelet counts, by means of a comparison with healthy controls. This case-control study included 19 patients newly diagnosed with ITP—whose blood samples were collected at four points in time: before any therapy and 1, 3, and 6 months after diagnosis—and 19 healthy controls. Tregs (CD4^+^ CD25^+^Foxp3 T cells) were evaluated by flow cytometry. There was a statistically significant difference in platelet count between the case and control groups. There were no significant differences in Treg counts between cases and controls at any point during the course of the study and no difference in Treg counts between the chronic and nonchronic groups and no significant correlation between Tregs and platelet counts in the case and control groups. The findings of this study did not show any statistically significant correlation between Tregs and number of platelets in the case and control groups. Treg cells did not play a role in the regulation of autoimmunity in children with ITP.

## 1. Introduction

Immune thrombocytopenic purpura (ITP) is an immune-mediated hemorrhagic condition that affects approximately 1 in 25,000 children every year [1, 2] and may present both as an acute, self-limiting condition and as a recurrent (chronic) form. ITP is characterized by premature destruction and clearance of platelets by mononuclear phagocytes [3]. Approximately 75% of patients recover spontaneously within 4 to 6 months [4]. Most children with ITP are previously healthy and are in fact the victims of their own imbalanced defenses [5].

Over the last two decades, several investigators have contributed to an improved understanding of the pathophysiology of this condition in an attempt to develop an individualized treatment approach for affected patients.

The ability to distinguish between self- and non-self-antigens is known as immune tolerance and plays an essential role in preventing intense self-recognition, which would produce pathological autoimmune responses [6, 7]. The term “peripheral tolerance” refers to mature reactive cells that escaped negative selection in the thymus and are suppressed from peripheral blood by a particular class of immunoregulatory cells, the regulatory T cells (Tregs) [4, 6, 8]. Tregs account for approximately 5% of circulating CD4^+^ T cells and are characterized by constitutive expression of transcription factor forkhead box protein 3 (Foxp3) molecules and high CD25 levels [9].

Recent studies have shown that patients with ITP exhibit antiplatelet self-reactive T cells and an imbalance in cytokine levels, which suggests loss of peripheral tolerance [10]. Regulatory T cell deficiency can lead to failure of peripheral tolerance and subsequent development of autoimmunity [11]. Furthermore, maintenance of immune tolerance and the effective immune response may be altered in the presence of an inflammatory process directed to target cells other than platelets [12].

The objective of this study was to evaluate the frequency of regulatory T cells in children with newly diagnosed ITP and the association between Tregs and platelet counts at the time of assessment, by means of a comparison with healthy controls.

The standardized terminology, definitions, and outcome criteria for ITP proposed by the International Work Group (IWG) shall be used throughout as follows: newly diagnosed ITP, within 3 months from diagnosis; persistent ITP, between 3 to 12 months from diagnosis; chronic ITP, lasting for more than 12 months; refractory ITP, failure to achieve remission after splenectomy and presence of severe ITP or risk of bleeding that requires therapy; and severe ITP, presence of major bleeding symptoms. Quality of response was defined as follows: complete remission (CR), platelet count ≥ 100,000/*μ*L and absence of bleeding; remission (R), platelet count ≥ 30,000/*μ*L, at least a twofold increase from the baseline count, and absence of bleeding; and nonremission (NR), platelet count < 30,000/*μ*L or less than a twofold increase from the baseline count or bleeding [13].

## 2. Methods

This prospective case-control study included 19 children between the ages of 1 and 15 years who were admitted to the pediatric emergency departments of two tertiary referral centers in Porto Alegre, RS, Brazil, between June 2010 and March 2012, due to clinical suspicion of ITP and thrombocytopenia (platelet count < 100,000/*μ*L). Patients with other autoimmune diseases, such as human immunodeficiency virus (HIV), systemic lupus erythematosus (SLE), and rheumatoid arthritis, were excluded from analysis. The minimum sample size had been calculated as 34 subjects (17 cases and 17 controls), for a significance level of 0.05, a statistical power of 90%, and a mean difference in Treg counts of 1.3 [9].

All patients were followed up for at least 6 months and underwent peripheral blood sampling for platelet counting and flow-cytometry-based quantification of Tregs. Samples were obtained at diagnosis and 1, 3, and 6 months later and designated sample 1, sample 2, sample 3, and sample 4, respectively. The control group was composed of retrospective blood samples that had been obtained from 19 healthy, age-matched children meeting the exclusion criteria described above. Children were identified by means of a chart review and only one sample was obtained from each control.

The study was approved by the research ethics committees of both centers where it was conducted, and the parents or legal guardians of all patients provided written informed consent for participation.

Blood samples were analyzed for Tregs by means of the following procedure: peripheral blood mononuclear cells (PBMCs) were isolated by density gradient centrifugation for 30 min at 900 g. In order to evaluate specific Treg subsets, cells were stained for 30 min with combinations of the following monoclonal antibodies: anti-CD4 FITC, anti-CD25 PE-CY7, and anti-Foxp3 PE (BD Biosciences, San Jose, CA, USA). Cells were acquired in the flow cytometer FACSCalibur (BD Biosciences, San Jose, CA, USA) by CellQuest Program.

Statistical analyses were performed with the Mann-Whitney *U* test for comparison of Tregs and platelet counts in the case and control groups, comparison of Tregs and platelet counts in the chronic and nonchronic subgroups, and comparison of Tregs in patients with platelet counts < 100,000/*μ*L and >100,000/*μ*L. The Friedman test was used for comparison between Tregs and platelet counts at the four time points determined for sampling. The Spearman correlation coefficient was used to assess the strength of association between Treg and platelet counts.

## 3. Results

Nineteen patients were assessed at the time of diagnosis, with collection of blood samples prior to any pharmacological therapy (sample 1). All four samples were collected in a timely manner (on diagnosis and at 1 month, 3 months, and 6 months) from 15 patients. Four patients withdrew from the study before all samples could be collected.

Mean age at diagnosis of ITP was 6.53 ± 4.14 years (median: 6.33 years). Eight patients were between the ages of 1 year and 4 years and 11 months, seven patients were between the ages of 5 years and 9 years and 11 months, and four were between the ages of 10 years and 15 years. The vast majority of patients (79%) were between the ages of 1 and 10 years at the time of diagnosis. Most patients in the case group (63%) were females.

Two patients achieved spontaneous remission of thrombocytopenia without any treatment. Of the 17 treated patients, 11 received a 2- to 3-week course of oral corticosteroids and two received corticosteroids for over 4 weeks. Four patients received both corticosteroids and intravenous immunoglobulin (IVIG) in combination at some point during treatment; three of these patients later required splenectomy due to chronic ITP. No patients received IVIG as monotherapy.

Of the 19 patients recruited at the start of the study, 14 achieved CR, two achieved R, and three progressed to chronic ITP. Of these, two achieved remission after splenectomy and only one developed refractory disease.

An overall analysis of the variables of interest (Tregs and platelet counts) is shown in Table 1. 

There was a statistically significant difference in platelet counts between the case and control groups in samples 1 and 4. There were no statistically significant differences in Treg counts between the case and control groups at any point in time (*P* > 0.05).

The Spearman correlation coefficient was used to assess the strength of association between Treg and platelet counts in the case and control groups as measured in samples 1, 2, 3, and 4. These data are shown in Table 2. There was no significant correlation between Treg and platelet counts in the case and control groups (*P* = 0.373), nor in the case or control groups when analyzed separately (*P* = 0.997 and *P* = 0.918, resp.).

The case group was further stratified into chronic (*n* = 3) and nonchronic patients (*n* = 16). The Mann-Whitney *U* test was used for comparison of Treg and platelet counts between these two subgroups. Results are shown in Table 3. There were no statistically significant differences in Treg counts between the chronic and nonchronic subgroups (*P* > 0.05). There was, however, a significant difference between platelet counts in samples 3 (obtained 3 months after diagnosis) and 4 (obtained 6 months after diagnosis) (*P* = 0.007 and *P* = 0.004, resp.).

Treg and platelet counts measured in all four samples (Treg1, Treg2, Treg3, and Treg4 and Platelet 1, Platelet 2, Platelet 3, and Platelet 4, resp.) were pooled for a total of 85 measurements.

This new subgroup was then stratified by platelet count ≤ 100.000/*μ*L (group 1) or >100.000/*μ*L (group 2)—in an attempt to detect any clinically relevant associations. The Mann-Whitney *U* test was used for a between-group comparison of Treg counts. There were no significant differences between Treg counts in patients with platelet counts of >100.000/*μ*L or ≤100.000/*μ*L (data not shown).

## 4. Discussion

Although the role of regulatory T cells in immune tolerance in healthy individuals appears to be well established, which would lead to the hypothesis that failure of the regulatory T cell system might induce autoimmunity [4, 6–8, 10, 14], the role of Tregs in childhood ITP has yet to be fully elucidated. 

Studies published over the last 10 years have reported discordant results as to the potential association between Treg and platelet counts, both in adults and in children, and in acute and chronic ITP alike.

In 2006, Liu et al. published a study in which adult patients with acute ITP or nonremission exhibited significantly lower relative Treg counts as compared with patients who had achieved remission, with no significant difference between the Treg counts of patients in remission and those of healthy controls [15]. Also in 2006, Fahim and Monir concluded that relative Treg counts were significantly lower in pediatric patients with acute and chronic ITP as compared with healthy controls, that patients with chronic ITP and platelet counts > 100,000/*μ*L had higher Treg counts than patients with thrombocytopenia (<100,000/*μ*L), and that corticosteroid-responsive patients also had higher Treg counts than nonresponsive patients [16].

In 2007, Sakakura et al. reported wide variation in Treg counts among adult ITP patients; in those with low platelet counts, there was no significant reduction in the number of Tregs. However, Treg counts were significantly elevated in patients with platelet counts >100,000/*μ*L as compared with healthy controls. These findings suggested that Tregs play an important role in platelet count recovery. It bears stressing that only seven of the 44 patients included in the study were newly diagnosed; all others had chronic ITP [17]. Yu et al. found no statistically significant difference in the frequency of CD4^+^ CD25^(hi)^Foxp3^+^ Tregs in adult patients with chronic ITP and in controls [18].

In the present study, there was no significant difference between Treg and platelet counts in the case and control groups (*P* = 0.373) and no significant correlation between Treg and platelet counts in either group on separate analysis (*P* = 0.997 and *P* = 0.918, resp.). The objective of the study was to assess the frequency of Tregs in patients with newly diagnosed ITP and ascertain whether an association exists between this frequency and platelet counts. No such association was found on diagnosis or in blood samples collected 1 month, 3 months, and 6 months after diagnosis. These findings do not contribute to the identification of potential predictors of progression to chronic ITP, nor do they advance the search for individualized treatment of specific cell deficiencies in ITP patients. Most probably, mechanisms other than Tregs are involved in the pathogenesis of autoimmunity in childhood ITP.

The population of this study was well defined in terms of age range (1 to 15 years) and, particularly, in terms of newly diagnosed status. At the time of initial blood collection, none of the patients in the case group were receiving any form of therapy that might have masked actual Treg cell counts. There are no data on the potential interference of corticosteroids and IVIG, for instance, on Treg count recovery in the short and long term. As yet, there is no way of determining whether Treg cell counts measured in patients with chronic or nonremitted ITP who are undergoing active treatment are representing or over estimating or underestimating actual counts. There is also no way of establishing whether expression of Tregs in adults can be extrapolated to pediatric populations or vice-versa.

Tregs (CD4^+^ CD25^+^Foxp3 T lymphocytes) have been described as immunoregulatory cells capable of suppressing self-reactive cells that escape negative selection in the thymus. If activated, these cells are liable to trigger autoimmunity. Quantitative failure of Tregs would justify this hypothesis. However, in this study, there was no significant association between Tregs and platelet counts in the case group or control group (*P* = 0.373), as described by some authors in recent years [15–17]. The possibility remains that a qualitative rather than quantitative failure of Tregs might trigger autoimmunity in children who develop ITP; that is, the function as well as the quantity of Tregs might be affected in children with ITP, thus potentially jeopardizing the regulation of peripheral tolerance. Conversely, another yet unknown mechanism may account for the development of autoimmunity in children. As this study did not confirm the hypothesis that Treg counts would be decreased in children with acute, chronic, or refractory ITP, further research on the pathophysiology and pathogenesis of this condition is required.

## Figures and Tables

**Table 1:** Comparison of Treg and platelet counts in the case and control groups.

Variable	Case group	Control group	*P* value*
Treg1 (*n* = 19)	0.11 (0.06–0.26)	0.22 (0.01–0.40)	0.908
Platelet 1 (*n* = 19)	12,473 (4,000–13,000)	300,000 (240,000–375,000)	<0.0001
Treg2 (*n* = 16)	0.13 (0.05–0.31)	0.22 (0.01–0.40)	0.987
Platelet 2 (*n* = 16)	215,687 (91,250–338,750)	300,000 (240,000–375,000)	0.076
Treg3 (*n* = 16)	0.13 (0.02–0.23)	0.22 (0.01–0.40)	0.659
Platelet 3 (*n* = 16)	215,812 (81,000–348,000)	300,000 (240,000–375,000)	0.066
Treg4 (*n* = 15)	0.10 (0.03–0.16)	0.22 (0.01–0.40)	0.410
Platelet 4 (*n* = 15)	248,000 (113,000–315,000)	300,000 (240,000–375,000)	0.033

Platelet counts expressed as median (interquartile range) and Treg counts as percentages.

*Mann-Whitney *U* test.

**Table 2:** Coefficients of correlation between Treg and platelet counts.

Variable	Correlation coefficient	*P* value
Cases + controls, Treg1, and platelet 1 (*n* = 38)	0.149	0.373
Controls, Treg1, and platelet 1 (*n* = 19)	−0.025	0.918
Cases, Treg1, and platelet 1 (*n* = 19)	0.001	0.997
Cases, Treg2, and platelet 2 (*n* = 16)	0.108	0.535
Cases, Treg3, and platelet 3 (*n* = 16)	0.020	0.911
Cases, Treg4, and platelet 4 (*n* = 15)	0.131	0.460

**Table 3:** Comparison between Treg and platelet counts in the chronic and nonchronic ITP subgroups.

Variable	Chronic	Nonchronic	*P* value*
Treg1 (*n* = 19)	0.09 (0.02–0.11)	0.12 (0.06–0.33)	0.254
Platelet 1 (*n* = 19)	5,000 (4,000–13,000)	6,000 (4,000–13,000)	0.793
Treg2 (*n* = 16)	0.10 (0.04–0.14)	0.15 (0.05–0.36)	0.364
Platelet 2 (*n* = 16)	136,000 (22,000–256,000)	252,000 (104,500–341,500)	0. 364
Treg3 (*n* = 16)	0.10 (0.06–0.73)	0.16 (0.02–0.22)	0.704
Platelet 3 (*n* = 16)	24,000 (15,000–81,000)	275,000 (138,000–370,500)	0.007
Treg4 (*n* = 15)	0.08 (0.03–0.10)	0.11 (0.04–0.21)	0.448
Platelet 4 (*n* = 15)	21,000 (17,000–30,000)	254,500 (184,250–316,500)	0.004

Platelet counts expressed as median (interquartile range) and Treg counts as percentages.

*Mann-Whitney *U* test.
